# Contribution of atrial myofiber architecture to atrial fibrillation

**DOI:** 10.1371/journal.pone.0279974

**Published:** 2023-01-31

**Authors:** Roya Kamali, Eugene Kwan, Misha Regouski, T. Jared Bunch, Derek J. Dosdall, Ed Hsu, Rob S. Macleod, Irina Polejaeva, Ravi Ranjan

**Affiliations:** 1 Department of Bioengineering, University of Utah, Salt Lake City, Utah, United States of America; 2 Cardiovascular Medicine, University of Utah, Salt Lake City, Utah, United States of America; 3 Nora Eccles Harrison Cardiovascular Research and Training Institute, Salt Lake City, Utah, United States of America; 4 Department of Animal, Dairy and Veterinary Sciences, Utah State University, Logan, Utah, United States of America; 5 Department of Surgery, University of Utah, Salt Lake City, Utah, United States of America; Universiteit Gent, BELGIUM

## Abstract

**Background:**

The role of fiber orientation on a global chamber level in sustaining atrial fibrillation (AF) is unknown. The goal of this study was to correlate the fiber direction derived from Diffusion Tensor Imaging (DTI) with AF inducibility.

**Methods:**

Transgenic goats with cardiac-specific overexpression of constitutively active TGF-β1 (n = 14) underwent AF inducibility testing by rapid pacing in the left atrium. We chose a minimum of 10 minutes of sustained AF as a cut-off for AF inducibility. Explanted hearts underwent DTI to determine the fiber direction. Using tractography data, we clustered, visualized, and quantified the fiber helix angles in 8 different regions of the left atrial wall using two reference vectors defined based on anatomical landmarks.

**Results:**

Sustained AF was induced in 7 out of 14 goats. The mean helix fiber angles in 7 out of 8 selected regions were statistically different (P-Value < 0.05) in the AF inducible group. The average fractional anisotropy (FA) and the mean diffusivity (MD) were similar in the two groups with FA of 0.32±0.08 and MD of 8.54±1.72 mm^2^/s in the non-inducible group and FA of 0.31±0.05 (P-value = 0.90) and MD of 8.68±1.60 mm^2^/s (P-value = 0.88) in the inducible group.

**Conclusions:**

DTI based fiber direction shows significant variability across subjects with a significant difference between animals that are AF inducible versus animals that are not inducible. Fiber direction might be contributing to the initiation and sustaining of AF, and its role needs to be investigated further.

## Introduction

Mechanisms perpetuating atrial fibrillation (AF), which is the most common clinical arrhythmia, are incompletely understood [1, 2]. AF is initiated by triggers with multiple theories to explain how it is sustained as it progresses to more advanced subtypes. One of these theories is that persistent AF is maintained by the propagation of multiple wavelets in the atria. Another theory is founded upon atrial re-entrant circuits and fibrillatory waves that drive AF progression [3–7]. Heterogeneity in electrophysiological properties and substrate provide the landscape for multiple wavelet reentry [8, 9].

The relevance of myocardial fiber orientation to the initiation and maintenance of cardiac arrhythmias like AF has not been comprehensively studied. Atrial fiber architecture and orientation are thought to be largely preserved across human subjects, although the orientation of the major fiber bundles may vary [10]. Fiber orientation defines the main direction for the propagation of excitation wave with the propagation being much faster parallel to the fiber direction as compared to the perpendicular direction [11]. AF progression with endocardial-epicardial dissociation has been shown to be related to differences in endocardial and epicardial fiber orientations and their rearrangements [12, 13]. Hence, there remain considerable gaps in our current understanding of fiber direction and arrhythmia genesis and progression; thus, a similar fiber orientation is often assigned to all the atrial arrhythmia computational models that do not differentiate between normal heart fiber orientation and fiber orientations in a heart with different types of arrythmia [14–16]. Only in a recent study, the effect of fiber orientation on the number of AF drivers and their location has been shown [17].

Most of the information about atrial fiber orientation is obtained from histological observations through sectioning the atria [18]. Creating the 3D fiber orientation data based on 2D sections can include bias and inaccuracies, and it is difficult to cover the whole chamber in detail based on a limited number of sections sampled from various regions of the atria.

Diffusion tensor imaging (DTI) has been used as a non-destructive tool for ex vivo imaging of the fiber orientation, which uses the restricted diffusivity of water molecules to assess the microstructure of the tissue. Using DTI, mean diffusivity (MD) of water molecules and directional variability of the water diffusion quantified by fractional anisotropy (FA) are measured. FA is a scalar measure between 0 (showing fully isotropic diffusion) to 1 (showing fully anisotropic diffusion). MD describes the rotationally invariant magnitude of water diffusion within the tissue, so an increase in MD normally indicates increased water content (due to inflammation or edema for example).The principal diffusion eigenvector in DTI gives the mean intravoxel orientation of the fiber. DTI has been used for different body organs, especially for ventricular fiber assignment measures, and recently some studies have used it for evaluating atrial fiber direction [10, 19]. The ventricular fiber orientations have been verified by histology based measured angles [20, 21]. DTI has been used on ventricles for detecting regional variations in cardiac tissue microstructure and its remodeling during disease [22]. The thin atrial wall makes the DTI acquisition more difficult, requiring higher resolution and quality compared to ventricular wall imaging techniques.

In this study, we investigated the effect of fiber direction in different left atrial regions on AF inducibility in TGF- β1 overexpressing transgenic goat models [23]. The TGF- β1 transgenic goat model was selected as a large animal model as it has susceptibility towards progressive atrial myopathy and AF [24].

## Methods

### AF inducibility in the TGF- β1 overexpressing goat model

We selected TGF- β1 overexpressing transgenic goats (n = 14) as a model of AF as reported earlier [23]. These studies were done under general anesthesia and after proper Institutional Animal Care and Use Committee (IACUC) approval at University of Utah and Utah State University. IACUC approval number at the University of Utah was 14–12010 and 17–11005. IACUC approval number at the Utah State University was 11026. General anesthesia was used for the electrophysiology studies. A combination cocktail of intravenous Ketamine and Diazepam was given for induction. Then the animals were intubated and anesthesia maintained with Isoflurane dosed to effect (2–4%) in inspired oxygen. Animals received flunixin meglumine (a NSAID) at a dose of 1.1 mg/kg IV immediately post-operatively to alleviate any suffering. Animals were monitored and if displayed evidence of additional pain or inflammation additional doses could be provided up to twice daily. AF inducibility was checked with electrophysiology experiments by rapid pacing them from a minimum of 3 different locations in the right atrium. Right atrial access was obtained by accessing the right jugular vein and placing a 6 Fr sheath. A deflectable quadripolar catheter was advanced to the right atrium under fluoroscopic guidance and was moved to different locations for the inducibility study. We paced the animal models with S1-S2 pacing trains at an S1 cycle length of 400ms and a single S2 stimulus with a decreasing coupling interval from 300 down to 200 in 20ms decrements and from 200ms in 10ms decrements until the atrial effective refractory period was reached or AF was induced as seen on the 12-lead surface ECG as well as the intracardiac signals. We also performed rapid burst pacing in the RA at 50Hz for 10 seconds at least three times if the S1-S2 pacing did not induce AF. The animals were determined to be inducible if they remained in AF for more than 10 minutes; if not, they were categorized as not inducible. We have included a summary of inducibility study and imaging dates in addition to the location of pacing and final S2 interval (ERP in not inducible animals) in supplementary materials. In these two groups, we compared the fiber orientation in 8 different regions of the left atrium to investigate the possible contribution of fiber orientation in the initiation and sustaining of AF.

### Preparing the hearts for imaging

The animals were euthanized using intravenous Beuthanasia and their hearts were extracted and imaged with an average of 107 days after the inducibility study as reported in supplementary material. After washing the hearts with sodium chloride and cleaning and cutting the fat around the heart, they were placed in cold phosphate-buffered saline (PBS). We then prepared the hearts for imaging by filling them with dental alginate mix through the pulmonary veins and superior and inferior vena cava to keep the atrial geometry and shape intact and prevent collapsing of the atrial wall. We then soaked them in fomblin to get rid of any trapped bubbles. After taking them out of fomblin, we placed them in 4% buffered formalin and stored them at room temperature at least 24 hours prior to MR imaging. The hearts that were not imaged right away were filled with alginate and stored in 10% neutral buffered formalin for a week. After a week of fixation, the hearts were stored in PBS in a refrigerator.

### MR imaging

MRI acquisitions were done on a Bruker 7.1 T horizontal bore MRI scanner (Bruker Biospin Inc., Ettlingen, Germany). Each heart specimen was placed in a container filled with alginate mix to keep the heart in place. Conventional DTI acquisition was performed using a standard multi-slice diffusion-weighted spin-echo sequence with the following imaging parameters: 500/30 ms TR/TE with an in-plane resolution of 0.66 mm. The slice thickness was 0.94 mm. A total of 12 diffusion sampling directions were acquired. The total scan time for each specimen (just the atria) was approximately 12 hours.

In order to have an accurate segmentation of the atria, we also scanned the heart specimens using a 3D T1-weighted gradient-echo MRI with an isotropic resolution of 0.20 mm^3^ (TE = 5.6 ms, TR = 30 ms, and scan duration about 9 hours). These higher resolution scans were used for accurate segmentation of the left atrium.

### Segmentation of the T1-weighted MRI

**Fig 1:**
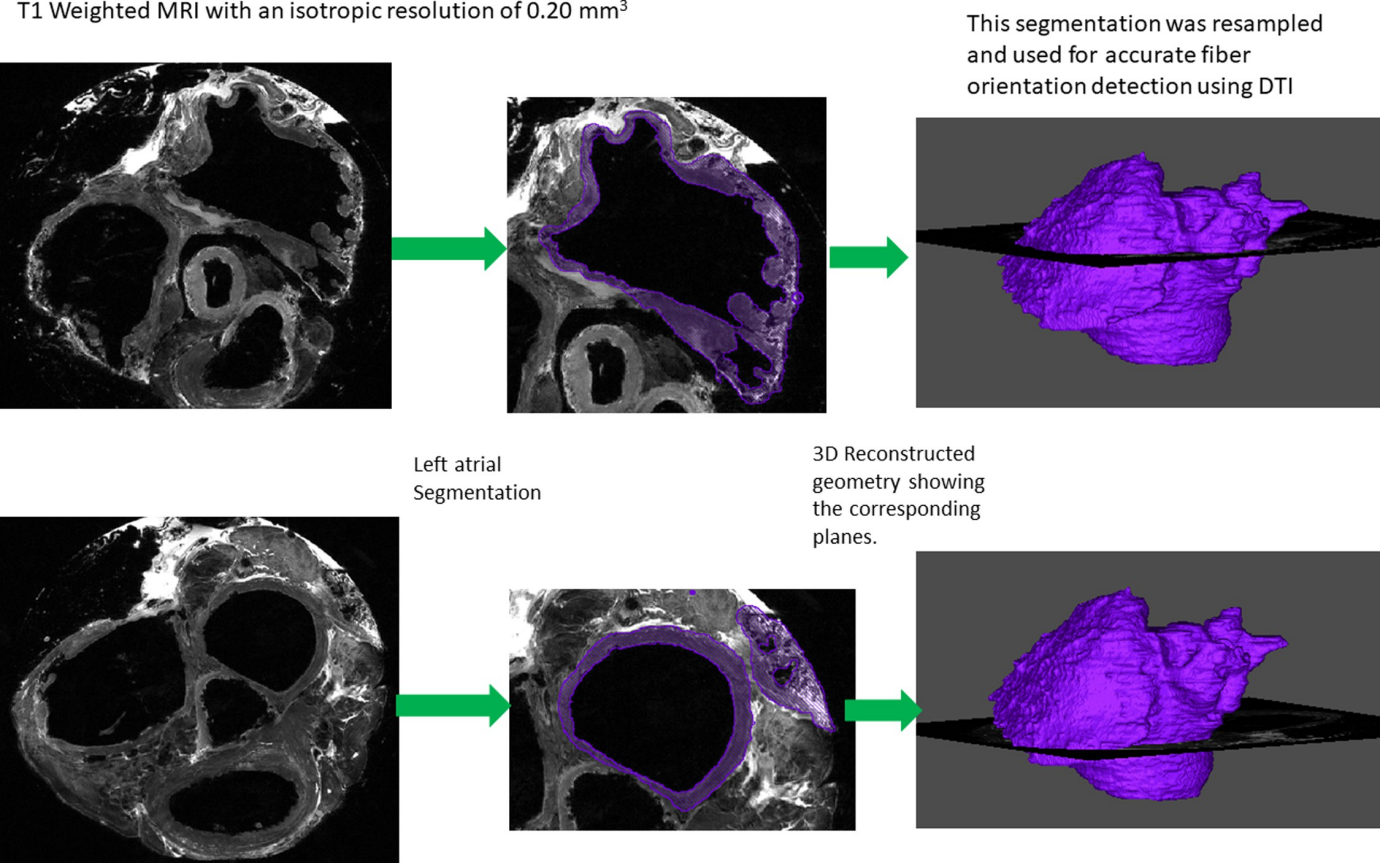
The segmentation of the high-resolution T1-weighted MRI used for the accurate fiber orientation detection using DTI.

The segmentation of the high-resolution MRI was done in Corview image processing software (University of Utah, Utah, USA). Using the Grow-Cut algorithm, the atrial wall was segmented [25], and a left atrial mask was created and resampled to match the resolution of the DTI scan. This mask was then used for accurate fiber tracking of the atrial wall as shown in Fig 1.

### Tractography

DSI studio (http://dsi-studio.labsolver.org) was used for visualizing the fiber directions. The diffusion tensor was calculated. A deterministic fiber tracking algorithm was used [26]. A seeding region was placed in the left atrium based on the mask created using LGE-MRI. The stopping criteria were angular threshold of 45 degrees and the anisotropy (FA) threshold of 0.065 as used in prior published reports for the atrial fiber tracking (10). The fiber trajectories (which we call tracts) were smoothed by averaging the propagation direction with a percentage of the previous direction. Tracts with a length shorter than 10 mm were discarded for better visualization. We visualized a total of 1,000,000 tracts for each goat model. FA and MD were also calculated using DSI studio for the segmented atrial walls.

### Fiber direction heterogeneity characterization

**Fig 2:**
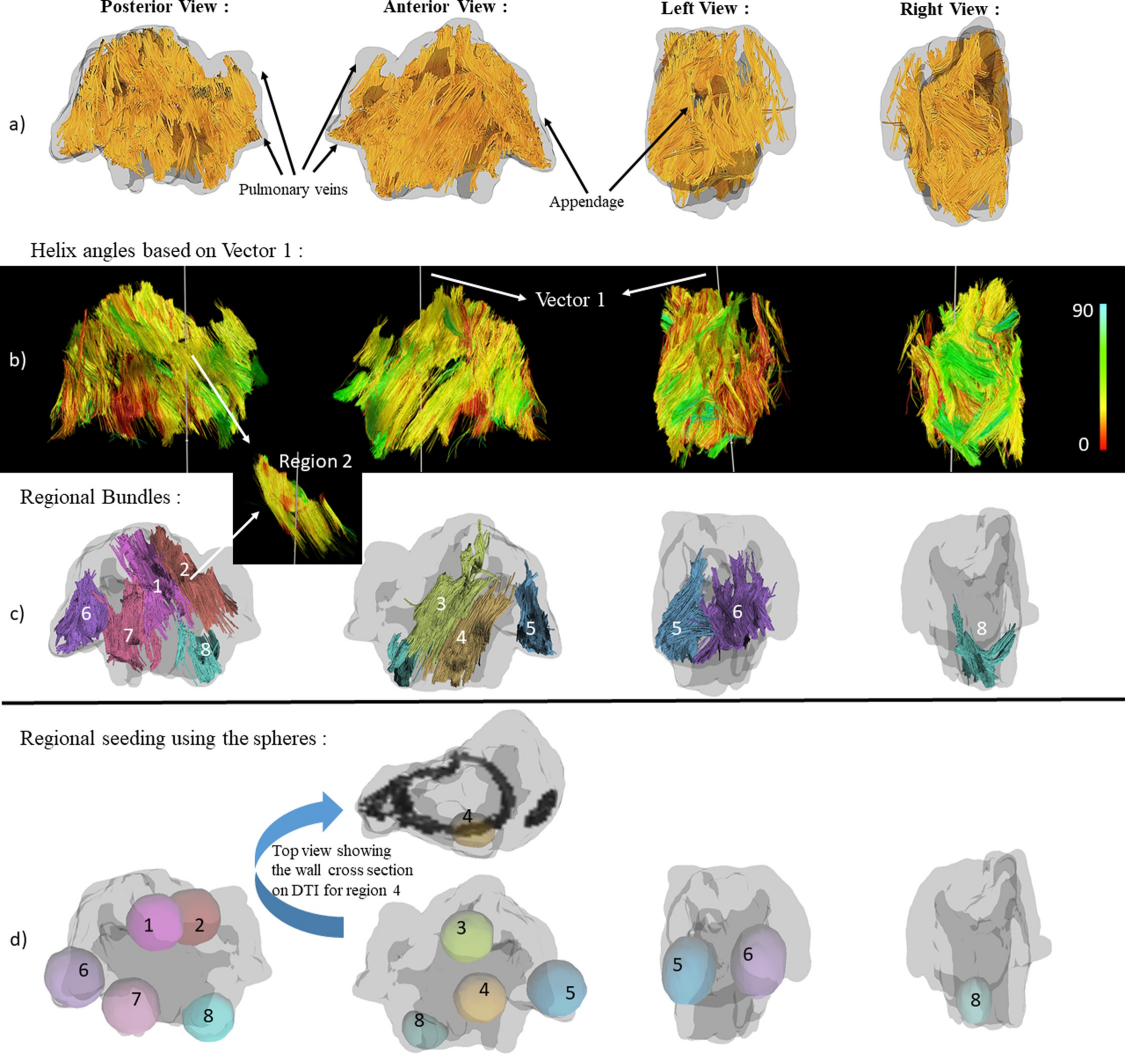
DTI based fiber orientation. a) Different views of the left atrium in one animal with the 3D fiber orientation before region selection. b) Fiber orientations color coded with helix angle (from 0 to 90 degree) with the vector perpendicular to the mitral valve plane (vector 1) chosen as the reference vector. c) Fibers in the eight different selected regions, each with a different color. d) Spheres chosen for regional seeding of the regions shown with different colors.

To characterize the heterogeneity in the fiber direction throughout the left atrium, 8 different regions were created based on anatomical landmarks, as shown in Fig 2C and 2D. The 8 regions were spatially distributed on the surface of the left atrium with region 1 and 2 on the posterior wall between the appendage and the pulmonary veins, region 3 and 4 on the anterior side of the left atrium between the appendage and pulmonary veins, region 5 and 6 on the appendage itself, region 7 on the posterior wall right next to the appendage and region 8 below the pulmonary veins. To create the regions, spheres with a diameter of 5 mm that covered the wall thickness were drawn to track the fibers going through them in each region as shown in Fig 2D. The fiber tracking parameters were the same as tracking parameters for the whole atrium with an additional limiting length factor. Tracts with a length shorter than 10 mm were discarded and only up to 20 mm of the length of the tracks inside or starting from the regions of interest were chosen to focus on the fiber direction in the selected regions only. A total of 10000 tracts were calculated for each specific region.

**Fig 3:**
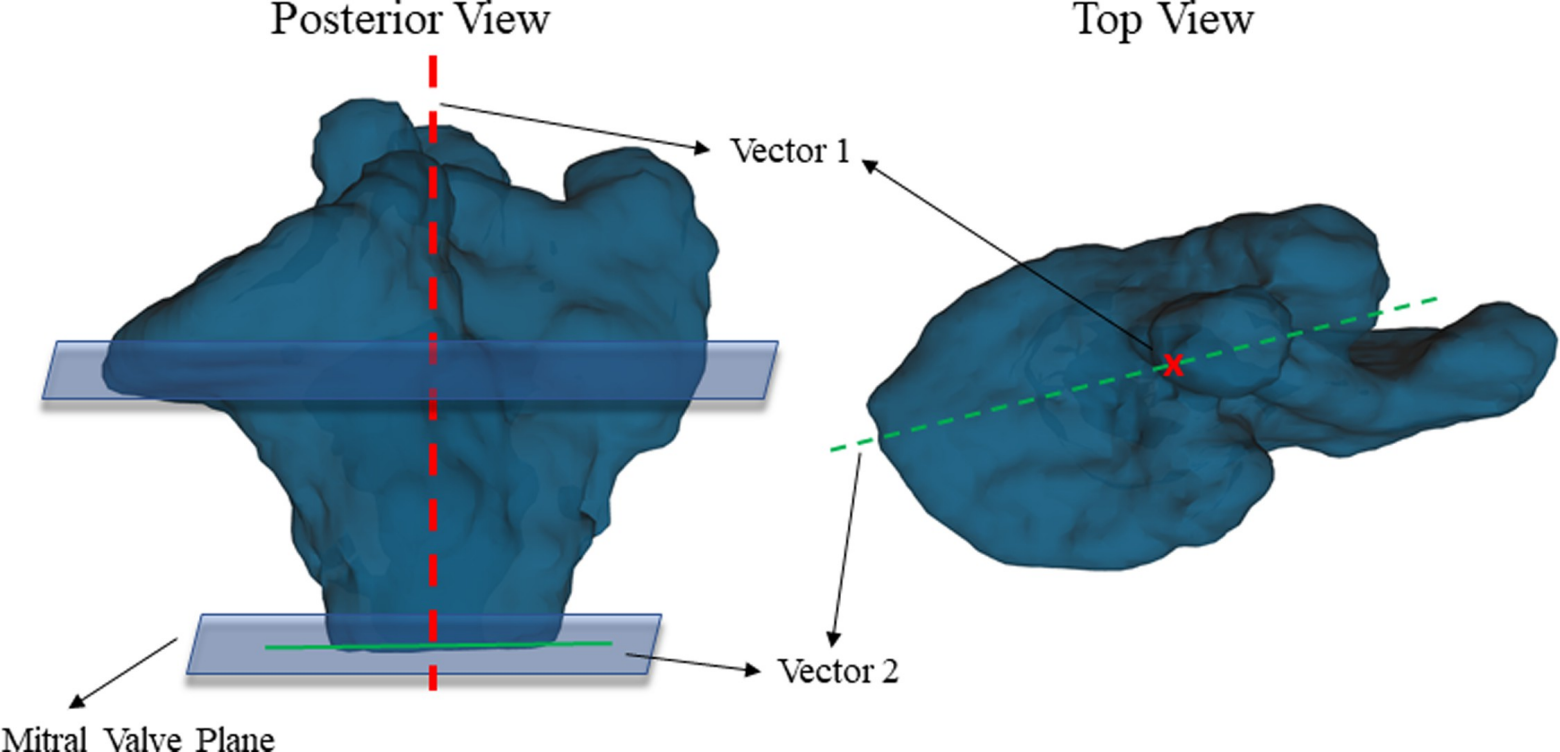
Reference vectors for comparing the fiber direction s between different regions of the left atrium and across different animals. Vector 1 (shown in red) is perpendicular to the mitral valve plane and vector 2 (shown in green) lays on the plane parallel to the mitral valve and starts from the midpoint between pulmonary veins to the tip of the appendage.

The tracts were exported from DSI studio and were imported into Trackvis [27] for further analysis. To compare the fiber direction between different animals, we defined two reference vectors that were consistent in all the animals. The two reference vectors were anatomy based; Vector 1 was defined as being perpendicular to the mitral valve plane pointing to the LA roof and vector 2 was defined in the plane passing through the mitral valve starting from the midpoint between pulmonary veins and pointing to the tip of the appendage when looking at that plane from the top, as shown in Fig 3.

The average fiber helix angles, along with their standard deviation (SD) in each of the selected regions, was measured by using the aforementioned reference vectors. Helix angle was defined by the inclination of the fiber after choosing the selected baseline vectors and was calculated using Trackvis as used in previous fiber direction studies [28, 29].

### Statistical analyses

Different variables are expressed as mean ± SD. Unpaired student t-test was used to compare the measured helix angles across the 8 selected regions between the AF inducible group and the non-inducible group using each reference vector separately.

## Results

We were able to induce AF in 7 out of 14 goats. The DTI acquisition resolution was 0.66 × 0.66 ×0.94 mm^3^. The average FA for all the segmented left atria was 0.31±0.07. The average MD for 14 animals was 8.61±1.66 ×10^−4^ mm^2^/s. In Table 1, FA and MD values for each animal along with the average values for both AF inducibility based groups are reported. The average for FA and MD for the non-inducible group was 0.32±0.08 and 8.54±1.72 mm^2^/s, and for the inducible group 0.31±0.05 (P-value = 0.90), and 8.68±1.60 mm^2^/s (P-value = 0.88), respectively, showing no significant difference in diffusion anisotropy pattern or the presence of inflammation or edema between the two groups.

**Table 1:** Fractional anisotropy (FA) and mean diffusivity (MD) values for all the animals along with the average values for each AF inducibility based group.

Inducible	FA	MD (mm^2^/s)	Not Inducible	FA	MD (mm^2^/s)
Goat 1	0.269	9.09	Goat 8	0.274	9.00
Goat 2	0.360	7.12	Goat 9	0.310	9.07
Goat 3	0.299	6.85	Goat 10	0.302	6.80
Goat 4	0.336	7.46	Goat 11	0.229	11.39
Goat 5	0.250	11.42	Goat 12	0.240	6.29
Goat 6	0.405	8.48	Goat 13	0.490	7.17
Goat 7	0.258	10.40	Goat 14	0.368	10.04
Average ± SD	0.31±0.05	8.68±1.60	Average ± SD	0.32±0.08	8.54±1.72

**Fig 4:**
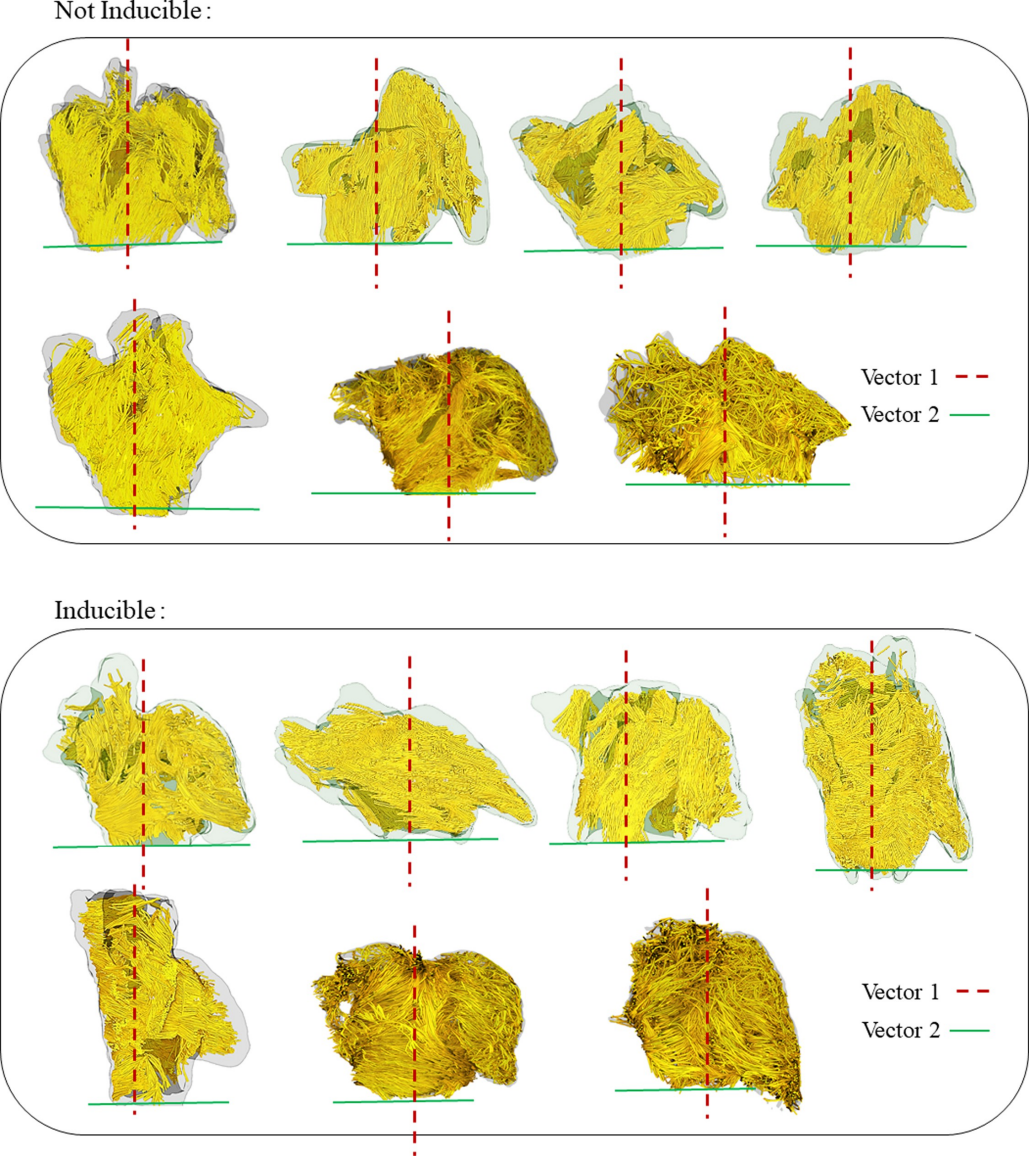
Anterior views of 3D fiber orientations in all scanned goats categorized based on their AF inducibility. The reference vector 1 is shown in red and reference vector 2 is shown in green.

**Fig 5:**
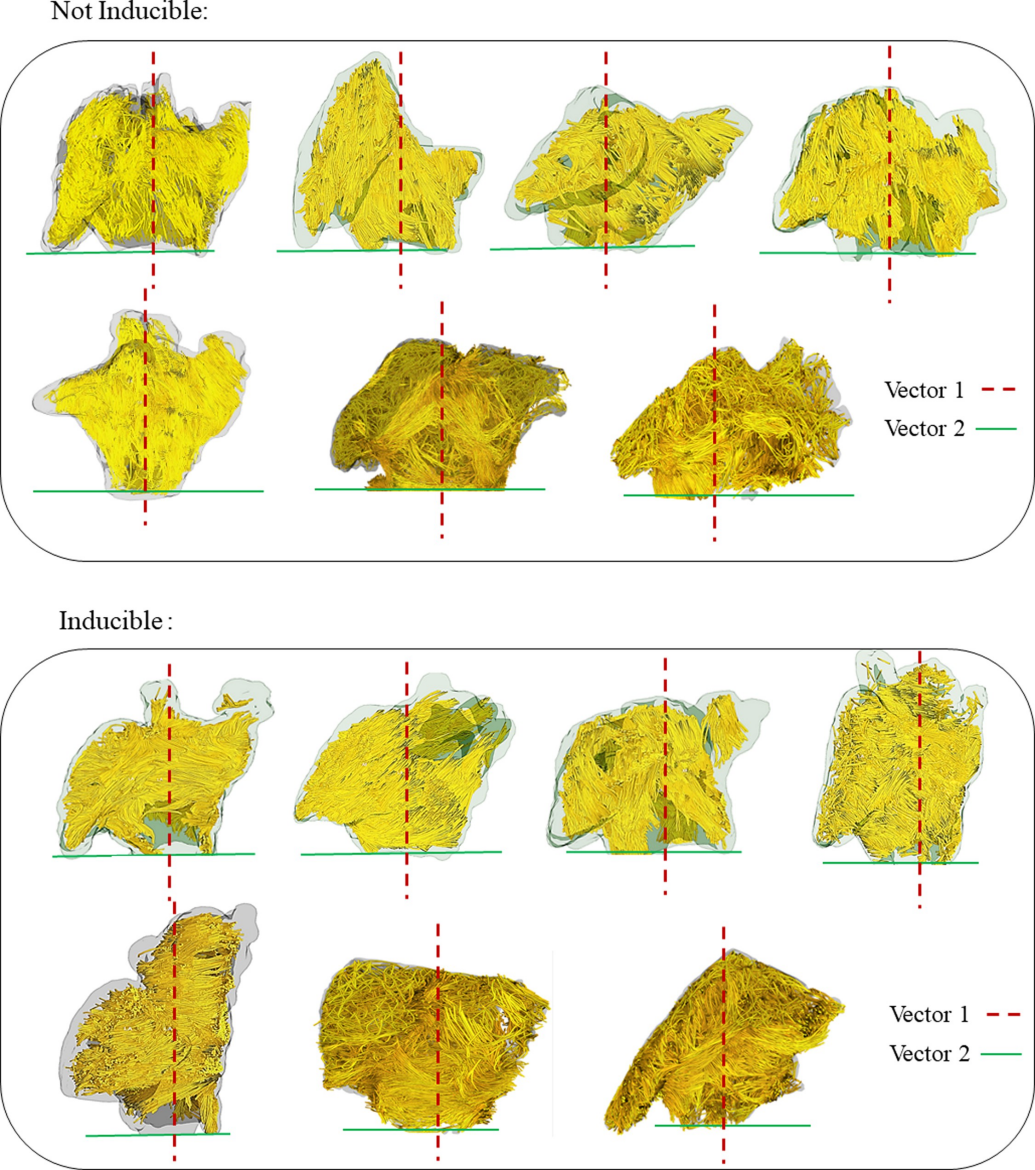
Posterior views of 3D fiber orientations in all scanned goats categorized based on their AF inducibility. The reference vector 1 is shown in red and reference vector 2 is shown in green.

The measured helix fiber angles for all the regions are reported in Tables 2 and 3 for AF inducible and non-AF inducible animals using vector 1 and vector 2 as a reference vector, respectively. Based on the reported p-values in Table 2, there is a significant difference between the reported helix angles in 5 out of 8 selected regions when choosing vector 1 as the reference vector. When choosing vector 2 as the reference, based on the reported p-values in Table 3, there is a significant difference between the reported helix angles in 7 out of 8 selected regions. Based on the reference vector 1, as shown in Fig 2B, larger helix angle by definition means that the fibers are positioned more vertically (with 0 degree being parallel with vector 1) and smaller helix angle means they are closer to being on a plane parallel to the mitral valve plane (with 90 degree being perpendicular to vector 1). Figs 4 and 5 show the anterior and posterior views of all the scanned goats categorized based on their AF inducibility, respectively. In these Figures, the atrial fibers are shown before the region selection along with the selected reference vectors for each of the caprine atria. Fig 2 shows the selected regions in an example of a non-inducible goat. The fiber orientation in the regions on the posterior and anterior walls that are away from the appendage or pulmonary veins and the regions on the right side of the appendage (regions 1, 2, 3, 6, and 7) are significantly different in these two groups as reported in Table 2. The angle indicates that the fibers are positioned more horizontally (perpendicular to vector 1) in the AF inducible group (significantly larger helix angles). Table 3 based on vector 2 as the reference, shows similar results with helix angles being significantly smaller in the AF inducible groups (similarly implying that they are positioned more horizontally) in regions 1,2,3,4,5,6 and 7. The regions on the anterior or posterior wall that are close to the appendage (region 4) or below the pulmonary veins (region 8) or on the appendage, region that are harder to accurately image (region 5) did not show significant differences in the two groups based on vector 1. However, based on vector 2, regions 4 and 5 were still significantly different leaving us with only one region (region 8) not showing a significantly different fiber orientation. We also checked the variation (SD) or heterogeneity of the measured helix fiber angles in the AF inducible versus non-inducible group as reported in Tables 2 and 3. The average regional standard deviations (SDs) were larger in the AF inducible group; however, calculated P-values for SDs in each region showed no significant difference between the two groups; no matter which vector was used as the reference.

**Table 2:** Measured average Helix angles in 8 regions with vector 1 chosen as the reference vector for the two AF inducibility based animal groups. P-values showing the difference in the helix angles between the two AF inducibility-based groups in each region are also reported.

**Inducible**	**Region 1**	**Region 2**	**Region 3**	**Region 4**	**Region 5**	**Region 6**	**Region 7**	**Region 8**
Goat 1	62 ± 15	64 ± 15	40 ± 10	32 ± 12	43 ± 35	84 ± 13	75 ± 14	51 ± 14
Goat 2	71 ± 10	80 ± 9	61 ± 21	69 ± 11	51 ± 15	81 ± 13	70 ± 19	33 ± 6
Goat 3	42 ± 15	38 ± 8	42 ± 13	39 ± 18	52 ± 16	35 ± 10	48 ± 22	66 ± 13
Goat 4	59 ± 16	49 ± 19	66 ± 10	45 ± 19	70 ± 17	71 ± 15	74 ± 10	81 ± 8
Goat 5	80 ± 9	81 ± 7	60 ± 17	67 ± 12	53 ± 13	61 ± 12	58 ± 18	68 ± 24
Goat 6	45 ± 13	46 ± 9	53 ± 11	70 ± 16	65 ± 10	41 ± 7	48 ± 19	69 ± 10
Goat 7	46 ± 11	42 ± 11	51 ± 25	35 ± 13	30 ± 21	65 ± 14	48 ± 14	44 ± 13
Average	58 ± 13	57 ± 12	53 ± 16	51 ± 15	52 ± 20	63 ± 12	60 ± 17	59 ± 14
**Not inducible**	**Region 1**	**Region 2**	**Region 3**	**Region 4**	**Region 5**	**Region 6**	**Region 7**	**Region 8**
Goat 8	30 ± 6	31 ± 7	36 ± 13	31 ± 5	64 ± 14	19 ± 7	28 ± 30	65 ± 13
Goat 9	30 ± 15	39 ±6	25 ± 8	29 ± 10	38 ± 15	17 ± 6	33 ± 12	12 ± 12
Goat 10	36 ± 8	38 ± 9	43 ± 9	32 ± 18	29 ± 15	42 ± 7	56 ± 13	35 ± 8
Goat 11	38 ± 10	34 ± 8	42 ± 10	33 ± 6	42 ± 15	18 ± 7	22 ± 8	62 ± 12
Goat 12	32 ± 8	37 ± 8	40 ± 12	32 ± 9	28 ± 9	33 ± 14	30 ±10	36 ± 12
Goat 13	46 ± 6	24 ± 5	49 ± 8	72 ± 9	58 ± 6	33 ± 11	60 ±11	56 ± 6
Goat 14	33 ± 14	41 ± 20	44 ± 8	49 ± 8	70 ± 5	33 ± 10	65 ± 16	55 ± 12
Average	35 ± 10	35 ± 10	40 ± 10	40 ± 10	47 ± 12	35 ± 9	42 ± 16	46 ± 11
P-value	0.005	0.016	0.015	0.222	0.552	0.018	0.049	0.200
P-value (SD)	0.052	0.105	0.102	0.053	0.218	0.117	0.438	0.509

**Table 3:** Measured average Helix angles in 8 regions with vector 2 chosen as the reference vector for the two AF inducibility based animal groups. P-values showing the difference in the helix angles between the two AF inducibility-based groups in each region are also reported.

**Inducible**	**Region 1**	**Region 2**	**Region 3**	**Region 4**	**Region 5**	**Region 6**	**Region 7**	**Region 8**
Goat 1	47 ± 13	53 ± 18	42 ± 10	59 ± 17	51 ± 18	43 ± 13	37 ± 5	34 ± 7
Goat 2	61 ± 5	58 ± 2	70 ± 9	65 ± 8	65 ± 4	63 ± 5	68 ± 8	75 ± 9
Goat 3	58 ± 12	56 ± 9	75 ± 11	73 ± 12	63 ± 15	49 ± 9	58 ± 10	82 ± 6
Goat 4	41 ± 4	42 ± 4	43 ± 4	40 ± 3	46 ± 9	45 ± 6	44 ± 6	44 ± 4
Goat 5	67 ± 12	61 ± 13	57 ± 15	65 ± 14	69 ± 13	65 ± 10	62 ± 14	51 ± 10
Goat 6	51 ± 14	54 ± 19	70 ± 19	35 ± 17	37 ± 10	56 ± 11	43 ± 19	74 ± 13
Goat 7	74 ± 14	63 ± 15	70 ± 12	68 ± 10	76 ± 11	66 ± 18	62 ± 18	64 ± 11
Average	57 ± 11	55 ± 13	60 ± 12	58 ± 12	58 ± 12	56 ± 11	53 ± 12	61 ± 9
**Not Inducible**	**Region 1**	**Region 2**	**Region 3**	**Region 4**	**Region 5**	**Region 6**	**Region 7**	**Region 8**
Goat 8	80 ± 8	76 ± 7	71 ± 11	71 ± 7	78 ± 10	78 ± 7	55 ± 15	81 ± 6
Goat 9	78 ± 12	77 ± 9	80 ± 6	79 ± 6	76 ± 10	81 ± 8	70 ± 14	83 ± 4
Goat 10	65 ± 15	72 ± 10	80 ± 8	76 ± 12	67 ± 21	64 ± 16	65 ± 13	65 ± 12
Goat 11	68 ± 11	73 ± 7	64 ± 11	68 ± 6	78 ± 8	74 ± 9	84 ± 6	77 ± 7
Goat 12	69 ± 10	63 ± 11	66 ± 9	71 ± 8	77 ± 10	74 ± 13	75 ± 9	76 ± 11
Goat 13	68 ± 16	65 ± 7	74 ± 7	59 ± 16	82 ± 7	80 ± 5	66 ± 17	50 ± 12
Goat 14	71 ± 7	65 ± 8	65 ± 4	62 ± 4	73 ± 9	67 ± 6	54 ± 6	78 ± 6
Average	71 ± 12	70 ± 9	71 ± 8	69 ± 9	76 ± 12	74 ± 10	67 ± 12	73 ± 9
P-Values	0.027	0.010	0.048	0.046	0.034	0.006	0.048	0.261
P-Value (SD)	0.750	0.349	0.172	0.305	0.862	0.634	0.777	0.898

## Discussion

In this study, we used caprine models with atrial fibrosis and AF susceptibility, and through EP stimulation studies checked their AF inducibility, finding the arrhythmia in 7 out of 14 animals. We reconstructed the 3D myofiber organization nondestructively using high-resolution DTI imaging of the hearts ex vivo and investigated the fiber orientations in 8 different left atrial regions based on anatomical landmarks to cover the whole atrial wall. Due to the complex geometry and the positioning of the atria, there was a need for defining a reproducible coordinate system. Coordinate systems have previously been defined for ventricles or atria based on the different study designs [10, 30]. We measured the mean helix fiber angles in each defined region with respect to our consistent anatomical based coordinate system. Finally, the comparisons made between the inducible and not inducible groups assessing the measured angles in each region showed significant differences in 7 out of 8 selected regions with the fibers being more horizontally positioned in the AF inducible group.

Prior studies have shown that complex fiber orientation and architecture are keys for normal sinus conduction, conduction velocities, and regional delays and blocks in the atria [31–33]. To our knowledge, this is the first study that has been done to look at the contribution of the myofiber structure and orientation to AF inducibility at the whole left atrial level and not just at a few specific locations. The roles of atrial myofiber structure and heterogeneous fiber orientation in rhythm disorders have previously been studied in specific regions [8, 12, 32, 34, 35]. For example, the role of changes in fiber orientation in septopulmonary regions, causing the sink to source mismatch and low safety for propagation, creating a substrate for atrial fibrillation, has been previously shown [35].

On a microscopic scale, conduction velocity has been shown to be anisotropic, with higher velocities along the parallel axis of the fiber and much slower velocities along the perpendicular axis of the fiber. The anisotropy ratio of signal propagation (longitudinal versus transverse with respect to fiber orientation) for isolated atrial muscle bundles of young individuals has been reported to be 4.5, which has been shown to increase two folds for older patients [8, 36]. Modifying the input fiber field choice in AF simulations have shown changes both in the number of drivers and their location [17]. The observed changes in fiber direction in our study in the inducible animals seem to direct the activity more horizontally as shown by the reported helix angles, moving it along the atria more than in non-inducible animals where it is oriented more towards the ventricles. This may be a facilitator for re-entrant activity in the atria by giving the cells more time to get out of their refractory period and be able to get re-excited while the signal travels down the atrium vertically.

Computational models are being used for proposing patient-specific ablation patterns that terminate atrial flutter or AF [16, 37, 38]. The importance of the fiber orientation in the direction of conduction and conduction velocity makes the implementation of the correct fiber orientation in simulations crucial. However, currently, the similar atlases or Laplace-based algorithms for estimating fiber orientation based on anatomy are being used for both normal cases and cases with arrhythmia [39, 40]. Only a recent computational study takes advantage of a database of 8 different atrial fiber orientations and chooses the best fiber atlas resulting in realistic atrial activation during arrythmia; however this study doesn’t focus on the differences in fiber orientation in different left atrial regions that show AF inducibility [17]. Based on our study, the fiber orientation can differ significantly between inducible and non-inducible groups, so we need to be aware of this limitation in the current computational models and ideally move towards acquiring more patient-specific information. The current study used isolated hearts with imaging at 7T for determining such changes making it unsuitable for use in patients, unless a database gets created and later used based on ex-vivo imaging of multiple atria of deceased patients with AF or other cardiac arrythmia. The development of different DTI sequences for measuring patient-specific fiber orientation in vivo will increase the accuracy of simulation results in the future.

### Limitations

The resolution might not have been enough for detecting the changes in fiber direction across thin regions of the left atrial wall especially the appendage. A higher resolution might be needed for the detection of the fiber orientation changes from epicardium to endocardium. This imaging technique can only be used ex vivo for the atria at this time. Hence, there remains a need for fiber orientation detection and quantification in vivo to allow for practical application. This study was done on caprine models that are susceptible to AF, which needs to be verified on other species and especially for human hearts. We only investigated the fiber orientation on the left atrium, right atrium also needs to be investigated in future.

## Conclusion

Based on this study, fiber orientation in certain regions of left atrium plays a role in the initiation and sustaining of AF and it tends to be more horizontally positioned in the AF inducible group. This needs to be considered in planning for AF treatments, especially in patient-specific simulation-based ablation procedures.

## Supporting information

S1 Table Has the EP study and imaging dates in addition to the location of pacing and final S2 interval or ERP (in animals that were not inducible). (DOCX)

S1 File (GZ)

S2 File (GZ)

S3 File (GZ)

S4 File (GZ)

S5 File (GZ)

S6 File (GZ)

S7 File (GZ)

S8 File (GZ)

S9 File (GZ)

S10 File (GZ)

S11 File (GZ)

S12 File (GZ)

S13 File (GZ)

S14 File (GZ)

## Abbreviations

AF: Atrial fibrillation
LGE: Late gadolinium enhancement
MRI: Magnetic resonance imaging

## References

[1] Chou C-C, Chen P-S. New concepts in atrial fibrillation: mechanism and remodeling. Medical Clinics of North America. 2008;92(1):53–63. doi: 10.1016/j.mcna.2007.08.008 18060997 PMC2213884

[2] Allessie MA, Boyden PA, Camm AJ, Kléber AG, Lab MJ, Legato MJ, et al. Pathophysiology and prevention of atrial fibrillation. Circulation. 2001;103(5):769–77. doi: 10.1161/01.cir.103.5.769 11156892

[3] Nattel S. New ideas about atrial fibrillation 50 years on. Nature. 2002;415(6868):219. doi: 10.1038/415219a 11805846

[4] Jalife J, Berenfeld O, Mansour M. Mother rotors and fibrillatory conduction: a mechanism of atrial fibrillation. Cardiovascular research. 2002;54(2):204–16. doi: 10.1016/s0008-6363(02)00223-7 12062327

[5] Comtois P, Kneller J, Nattel S. Of circles and spirals: bridging the gap between the leading circle and spiral wave concepts of cardiac reentry. EP Europace. 2005;7(s2):S10–S20. doi: 10.1016/j.eupc.2005.05.011 16102499

[6] Moe G. Atrial fibrillation as a self sustaining arrhythmia independent of focal discharge. Am Heart J. 1959;58:59–70. doi: 10.1016/0002-8703(59)90274-1 13661062

[7] Kamali R, Kump J, Ghafoori E, Lange M, Hu N, Bunch TJ, et al. Area available for atrial fibrillation to propagate is an important determinant of recurrence after ablation. Clinical Electrophysiology. 2021;7(7):896–908. doi: 10.1016/j.jacep.2020.11.008 33640348 PMC9255558

[8] Spach MS, Miller W 3rd, Dolber PC, Kootsey JM, Sommer JR, Mosher CE Jr. The functional role of structural complexities in the propagation of depolarization in the atrium of the dog. Cardiac conduction disturbances due to discontinuities of effective axial resistivity. Circulation research. 1982;50(2):175–91. doi: 10.1161/01.res.50.2.175 7055853

[9] Dillon SM, Allessie MA, Ursell PC, Wit AL. Influences of anisotropic tissue structure on reentrant circuits in the epicardial border zone of subacute canine infarcts. Circulation research. 1988;63(1):182–206. doi: 10.1161/01.res.63.1.182 3383375

[10] Pashakhanloo F, Herzka DA, Ashikaga H, Mori S, Gai N, Bluemke DA, et al. Myofiber architecture of the human atria as revealed by submillimeter diffusion tensor imaging. Circulation: arrhythmia and electrophysiology. 2016;9(4):e004133. doi: 10.1161/CIRCEP.116.004133 27071829 PMC7035884

[11] Roberts DE, Hersh LT, Scher AM. Influence of cardiac fiber orientation on wavefront voltage, conduction velocity, and tissue resistivity in the dog. Circulation research. 1979;44(5):701–12. doi: 10.1161/01.res.44.5.701 428066

[12] Spach MS. Anisotropic structural complexities in the genesis of reentrant arrhythmias. Circulation. 1991;84(3):1447–50. doi: 10.1161/01.cir.84.3.1447 1884469

[13] Maesen B, Zeemering S, Afonso C, Eckstein J, Burton RA, van Hunnik A, et al. Rearrangement of atrial bundle architecture and consequent changes in anisotropy of conduction constitute the 3-dimensional substrate for atrial fibrillation. Circulation: Arrhythmia and Electrophysiology. 2013;6(5):967–75. 23969531 10.1161/CIRCEP.113.000050

[14] Tobón C, Ruiz-Villa CA, Heidenreich E, Romero L, Hornero F, Saiz J. A three-dimensional human atrial model with fiber orientation. Electrograms and arrhythmic activation patterns relationship. PloS one. 2013;8(2). doi: 10.1371/journal.pone.0050883 23408928 PMC3569461

[15] Krueger MW, Schmidt V, Tobón C, Weber FM, Lorenz C, Keller DU, et al., editors. Modeling atrial fiber orientation in patient-specific geometries: a semi-automatic rule-based approach. International Conference on Functional Imaging and Modeling of the Heart; 2011: Springer.

[16] Kamali R, Gillete K, Tate J, Abhyankar DA, Dosdall DJ, Plank G, et al. Treatment Planning for Atrial Fibrillation Using Patient-Specific Models Showing the Importance of Fibrillatory-Areas. Annals of Biomedical Engineering. 2022:1–14. doi: 10.1007/s10439-022-03029-5 35930093 PMC10440744

[17] Roney CH, Bendikas R, Pashakhanloo F, Corrado C, Vigmond EJ, McVeigh ER, et al. Constructing a human atrial fibre atlas. Annals of biomedical engineering. 2021;49(1):233–50. doi: 10.1007/s10439-020-02525-w 32458222 PMC7773625

[18] Ho S, Sánchez‐Quintana D. The importance of atrial structure and fibers. Clinical Anatomy: The Official Journal of the American Association of Clinical Anatomists and the British Association of Clinical Anatomists. 2009;22(1):52–63. doi: 10.1002/ca.20634 18470938

[19] Pashakhanloo F, Herzka DA, Mori S, Zviman M, Halperin H, Gai N, et al. Submillimeter diffusion tensor imaging and late gadolinium enhancement cardiovascular magnetic resonance of chronic myocardial infarction. Journal of Cardiovascular Magnetic Resonance. 2017;19(1):9. doi: 10.1186/s12968-016-0317-3 28122618 PMC5264305

[20] Kung GL, Nguyen TC, Itoh A, Skare S, Ingels NB Jr, Miller DC, et al. The presence of two local myocardial sheet populations confirmed by diffusion tensor MRI and histological validation. Journal of Magnetic Resonance Imaging. 2011;34(5):1080–91. doi: 10.1002/jmri.22725 21932362 PMC3195899

[21] Scollan DF, Holmes A, Winslow R, Forder J. Histological validation of myocardial microstructure obtained from diffusion tensor magnetic resonance imaging. American Journal of Physiology-Heart and Circulatory Physiology. 1998;275(6):H2308–H18. doi: 10.1152/ajpheart.1998.275.6.H2308 9843833

[22] Carruth ED, Teh I, Schneider JE, McCulloch AD, Omens JH, Frank LR. Regional variations in ex-vivo diffusion tensor anisotropy are associated with cardiomyocyte remodeling in rats after left ventricular pressure overload. Journal of Cardiovascular Magnetic Resonance. 2020;22:1–13. 32241289 10.1186/s12968-020-00615-1 PMC7114814

[23] Polejaeva IA, Ranjan R, Davies CJ, Regouski M, Hall J, Olsen AL, et al. Increased susceptibility to atrial fibrillation secondary to atrial fibrosis in transgenic goats expressing transforming growth factor‐β1. Journal of cardiovascular electrophysiology. 2016;27(10):1220–9. 27447370 10.1111/jce.13049 PMC5065395

[24] Regouski M, Galenko O, Doleac J, Olsen AL, Jacobs V, Liechty D, et al. Spontaneous Atrial Fibrillation in Transgenic Goats With TGF (Transforming Growth Factor)-β1 Induced Atrial Myopathy With Endurance Exercise. Circulation: Arrhythmia and Electrophysiology. 2019;12(11):e007499. 31707807 10.1161/CIRCEP.119.007499

[25] Zhu L, Kolesov I, Gao Y, Kikinis R, Tannenbaum A, editors. An effective interactive medical image segmentation method using fast growcut. MICCAI workshop on interactive medical image computing; 2014.

[26] Yeh F-C, Verstynen TD, Wang Y, Fernández-Miranda JC, Tseng W-YI. Deterministic diffusion fiber tracking improved by quantitative anisotropy. PloS one. 2013;8(11). doi: 10.1371/journal.pone.0080713 24348913 PMC3858183

[27] Wang R, Benner T, Sorensen AG, Wedeen VJ, editors. Diffusion toolkit: a software package for diffusion imaging data processing and tractography. Proc Intl Soc Mag Reson Med; 2007: Berlin.

[28] Taylor EN, Hoffman MP, Barefield DY, Aninwene GE, Abrishamchi AD, Lynch IV TL, et al. Alterations in multi‐scale cardiac architecture in association with phosphorylation of myosin binding protein‐C. Journal of the American Heart Association. 2016;5(3):e002836. doi: 10.1161/JAHA.115.002836 27068630 PMC4943261

[29] Mekkaoui C, Huang S, Chen HH, Dai G, Reese TG, Kostis WJ, et al. Fiber architecture in remodeled myocardium revealed with a quantitative diffusion CMR tractography framework and histological validation. Journal of Cardiovascular Magnetic Resonance. 2012;14(1):70. doi: 10.1186/1532-429X-14-70 23061749 PMC3506570

[30] Bayer J, Prassl AJ, Pashaei A, Gomez JF, Frontera A, Neic A, et al. Universal ventricular coordinates: A generic framework for describing position within the heart and transferring data. Medical image analysis. 2018;45:83–93. doi: 10.1016/j.media.2018.01.005 29414438

[31] De Ponti R, Ho SY, SALERNO‐URIARTE JA, Tritto M, Spadacini G. Electroanatomic analysis of sinus impulse propagation in normal human atria. Journal of cardiovascular electrophysiology. 2002;13(1):1–10. doi: 10.1046/j.1540-8167.2002.00001.x 11843475

[32] Hocini M, Ho SY, Kawara T, Linnenbank AC, Potse M, Shahet al. Electrical conduction in canine pulmonary veins: electrophysiological and anatomic correlation. Circulation. 2002;105(20):2442–8. 12021234 10.1161/01.cir.0000016062.80020.11

[33] Hamabe A, Okuyama Y, Miyauchi Y, Zhou S, Pak H-N, Karagueuzian HS, et al. Correlation between anatomy and electrical activation in canine pulmonary veins. Circulation. 2003;107(11):1550–5. doi: 10.1161/01.cir.0000056765.97013.5e 12654615

[34] Markides V, Schilling RJ, Yen Ho S, Chow AW, Davies DW, Peters NS. Characterization of left atrial activation in the intact human heart. Circulation. 2003;107(5):733–9. doi: 10.1161/01.cir.0000048140.31785.02 12578877

[35] Klos M, Calvo D, Yamazaki M, Zlochiver S, Mironov S, Cabrera J-A, et al. Atrial septopulmonary bundle of the posterior left atrium provides a substrate for atrial fibrillation initiation in a model of vagally mediated pulmonary vein tachycardia of the structurally normal heart. Circulation: Arrhythmia and Electrophysiology. 2008;1(3):175–83. doi: 10.1161/CIRCEP.107.760447 19609369 PMC2710853

[36] Spach MS, Dolber PC. Relating extracellular potentials and their derivatives to anisotropic propagation at a microscopic level in human cardiac muscle. Evidence for electrical uncoupling of side-to-side fiber connections with increasing age. Circulation research. 1986;58(3):356–71. doi: 10.1161/01.res.58.3.356 3719925

[37] Zahid S, Whyte KN, Schwarz EL, Blake RC III, Boyle PM, Chrispin J, et al. Feasibility of using patient-specific models and the “minimum cut” algorithm to predict optimal ablation targets for left atrial flutter. Heart rhythm. 2016;13(8):1687–98. doi: 10.1016/j.hrthm.2016.04.009 27108938 PMC5972526

[38] Boyle PM, Zghaib T, Zahid S, Ali RL, Deng D, Franceschi WH, et al. Computationally guided personalized targeted ablation of persistent atrial fibrillation. Nature biomedical engineering. 2019;3(11):870–9. doi: 10.1038/s41551-019-0437-9 31427780 PMC6842421

[39] Fastl TE, Tobon-Gomez C, Crozier A, Whitaker J, Rajani R, McCarthy KP, et al. Personalized computational modeling of left atrial geometry and transmural myofiber architecture. Medical image analysis. 2018;47:180–90. doi: 10.1016/j.media.2018.04.001 29753182 PMC6277816

[40] Zahid S, Cochet H, Boyle PM, Schwarz EL, Whyte KN, Vigmond EJ, et al. Patient-derived models link re-entrant driver localization in atrial fibrillation to fibrosis spatial pattern. Cardiovascular research. 2016;110(3):443–54. doi: 10.1093/cvr/cvw073 27056895 PMC4872878

