# Supplementary material for: Contribution of atrial myofiber architecture to atrial fibrillation
Source: PLoS One. 2023 Jan 31;18(1):e0279974. doi: 10.1371/journal.pone.0279974 (PMC9888724; doi:10.1371/journal.pone.0279974)
Supplement: S1 Table — (DOCX) [file pone.0279974.s001.docx]

| **Goat ID** | **EP Date** | **MRI date** | **final S2 interval**  **(ERP in not inducible)** | **Location of Pacing** |
| --- | --- | --- | --- | --- |
| Inducible |  |  |  |  |
| 1 | 8/17/2016 | 8/18/2016 | 140ms | RA free wall |
| 2 | 8/19/2016 | 8/20/2016 | 130ms | RAA |
| 3 | 9/30/2016 | 10/1/2016 | 130ms | High RA free wall |
| 4 | 10/12/2018 | 10/26/2018 | 90ms | RAA |
| 5 | 1/19/2018 | 2/28/2018 | 120ms | High RA free wall |
| 6 | 2/14/2019 | 2/20/2019 | 130ms | RAA |
| 7 | 1/30/2019 | 6/24/2020 | 90ms | RA free wall |
| Not Inducible |  |  |  |  |
| 8 | 8/3/2016 | 10/14/2016 | 180ms | High RA free wall |
| 9 | 8/5/2016 | 10/2/2016 | 150ms | RAA |
| 10 | 9/28/2016 | 9/29/2016 | 140ms | High RA free wall |
| 11 | 3/16/2018 | 3/23/2018 | 150ms | High RA free wall |
| 12 | 1/23/2019 | 7/11/2019 | 120ms | RA free wall |
| 13 | 2/27/2019 | 6/5/2020 | 120ms | RAA |
| 14 | 2/1/2019 | 7/12/2019 | 130ms | RA free wall |

Table 1. EP study and imaging dates in addition to the location of pacing and final S2 interval (ERP in not inducible animals).
